# Impact of Charge Transfer Complex on the Dielectric Relaxation Processes in Poly(methyl methacrylate) Polymer

**DOI:** 10.3390/molecules27061993

**Published:** 2022-03-19

**Authors:** Arwa Alrooqi, Zahra M. Al-Amshany, Laila M. Al-Harbi, Tariq A. Altalhi, Moamen S. Refat, A. M. Hassanien, A. A. Atta

**Affiliations:** 1 Department of Chemistry, Faculty of Sciences, King Abdulaziz University, P.O. Box 80203, Jeddah 21589, Saudi Arabia; asalemalroge@stu.kau.edu.sa (A.A.); zalamshany@kau.edu.sa (Z.M.A.-A.); lalhrbi@kau.edu.sa (L.M.A.-H.); 2 Department of Chemistry, Faculty of Arts and Science, Al-Baha University, P.O. Box 1988, Baljurashi 65634, Saudi Arabia; 3 Department of Chemistry, College of Science, Taif University, P.O. Box 11099, Taif 21944, Saudi Arabia; moamen@yahoo.com; 4 Department of Physics, College of Science and Humanities, Shaqra University, P.O. Box 1040, Al Quwaiiyah 11971, Saudi Arabia; ahassanien@su.edu.sa; 5 Department of Physics, College of Science, Taif University, P.O. Box 11099, Taif 21944, Saudi Arabia; a.atta@tu.edu.sa

**Keywords:** PMMA polymer, charge transfer complexes, relaxation processes

## Abstract

The impact of the charge transfer complex on the dielectric relaxation processes in free poly(methyl methacrylate) (PMMA) polymer sheets was investigated. The frequency dependence of dielectric properties was obtained over the frequency range 0.1 Hz–1 MHz at temperatures ranging between 303 K and 373 K for perylene dye and acceptors (picric acid (PA) and chloranilic acid (CLA)) in an in situ PMMA polymer. The TG/dTG technique was used to investigate the thermal degradation of the synthesized polymeric sheets. Additionally, the kinetic parameters have been assessed using the Coats–Redfern relation. The dielectric relaxation spectroscopy of the synthesized polymeric sheets was analyzed in terms of complex dielectric constant, dielectric loss, electrical modulus, electrical conductivity, and Cole–Cole impedance spectroscopy. α- and β-relaxation processes were detected and discussed. The σ(ω) dispersion curves of the synthesized polymeric sheets show two distinct regions with increasing frequency. The impedance data of the synthesized polymeric sheets can be represented by the equivalent circuit (parallel RC).

## 1. Introduction

The presently existing technologies based on inorganic semiconductor materials platforms suffer from rigidity and mechanical stiffness, which degrades the device performance. Polymers have received increasing attention in solution-processable technology due to their flexibility, transparency, and reduced weight. They have gained widespread consideration in recent years due to their valuable electronic, optoelectronic, electrochemical, and non-linear optical properties [1,2,3,4,5,6].

Poly(methyl methacrylate) or so-called acrylic glass is an electron donor material that can be used in the synthesis of solid polymeric materials. It has an amorphous structure due to the presence of the ester group in its backbone chain. PMMA is a polar dimensional stable thermoplastic material with high transparency and high strength [7]. On the other hand, the donor–acceptor interfaces of the small organic molecules exhibit smaller areas as well as higher crystallinity, which are opposed to charging transfer and exciton dissociation. Furthermore, polymer films with good molecular π–π stacking typically display high mobility for the electric charge carrier. The solar cells based on small organic molecules are characterized by a high open-circuit voltage (V_OC_) since it is mainly determined by the difference between the highest occupied molecular orbital (HOMO) of the donor molecule and the lowest unoccupied molecular orbital (LUMO) of the acceptor molecule. To improve electric charge generation and transport properties, the blend structure and surface morphology must also be enhanced [8,9,10].

The analysis of the dielectric relaxation characteristics of insulating polymers is difficult due to their dependency on backbone chain structure and impurities. The complex dielectric relaxation process is a powerful method for studying the molecular dynamics in a wide range of materials including glasses, oxides, and polymers. The wide range of frequencies allows investigations of various relaxation processes concurrently. The small changes in the dielectric properties of polymer as a response to the application of a frequency-dependent electric field can be measured with high accuracy [11,12,13,14]. Dielectric properties of hybrid PMMA/RGO/Fe_2_O_3_ nanocomposites fabricated by in situ polymerization increased the dielectric constant of PMMA [15]. The complex dielectric permittivities of polymer nanocomposites comprising a PMMA matrix and ZnO, SnO_2_, and TiO_2_ nanofillers (20 Hz to 1 MHz) were investigated by Sengwa and Dhatarwal [16]. They reported that the complex dielectric permittivities increased with the increase in the SnO_2_ and ZnO concentrations, whereas TiO_2_ loading lowered the permittivities as compared to the host PMMA matrix film. Dielectric characterization of polyvinyl chloride/poly(methyl methacrylate) was investigated [17]. The studied PVC/PMMA blends were found to be miscible for PMMA weight ratio less than or equal to 60%. An increase in the dielectric loss when the PMMA weight ratio increases was observed.

The *π*-conjugated organic materials that have alternating double bonds (as perylene dye) depict the mobility of electrons along the polymer backbone (delocalization) and usually lead to stabilization of the molecule. The strategy of the current research article is to explore the impact of the charge-transfer (CT) complexes of perylene dye, and acceptors (iodine, picric acid, and chloranilic acid) on the dielectric relaxation processes in in situ PMMA polymers. The frequency and temperature dependence of the dielectric constant and the electrical conductivity have been examined to discover and predict the electronic conduction mechanisms in the synthesized polymeric sheet samples.

## 2. Materials and Methods

**Figure 1:**
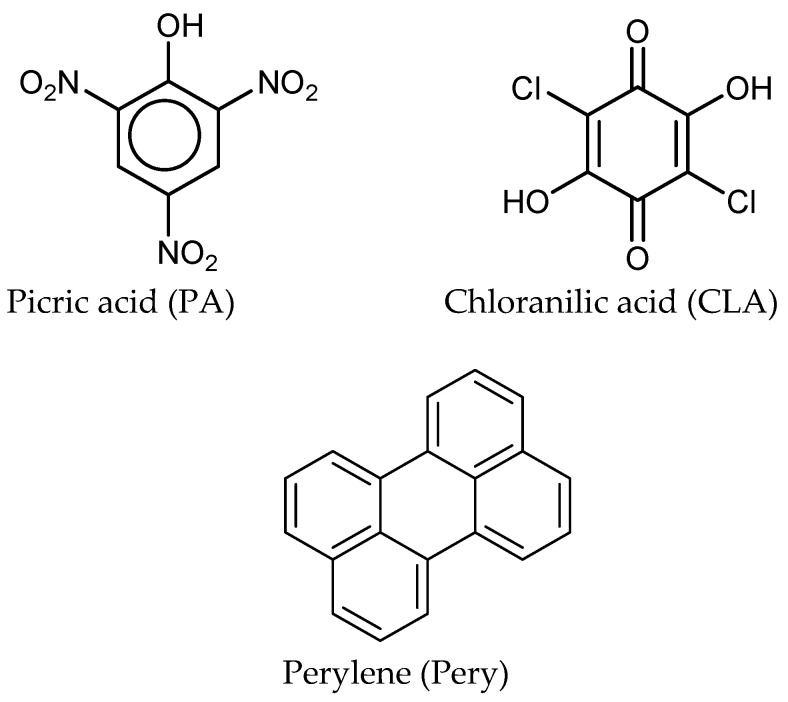

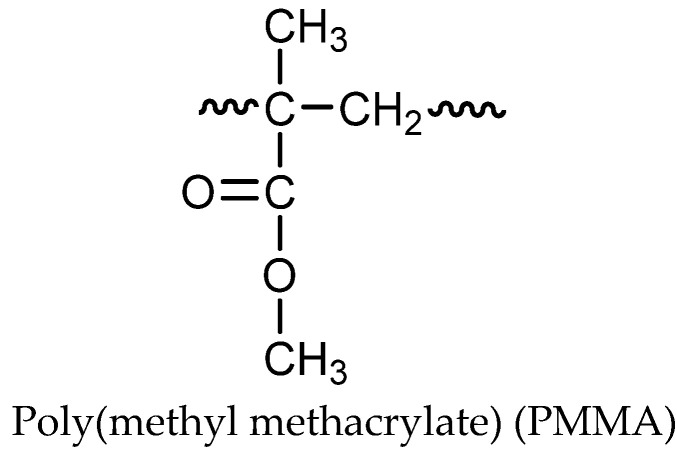
Chemical structures of PMMA, perylene donor and PA and CLA acceptors.

All chemicals used throughout this work were pure grade. Perylene (C_20_H_12_; ≥99%), and acceptors were purchased from Sigma-Aldrich. The chemicals were used as received. The general chemical structures of donor and acceptors are as follows (Figure 1):

The preparation of the charge-transfer (CT) complex of PMMA-perylene dye, PMMA-perylene-PA and PMMA-perylene-CLA polymer sheets was reported by Altalhi et al. [18].

Thermogravimetric analysis of the synthesized polymeric sheet samples in the temperature range of 303–873 K with a rate of 10 K min^−1^ was analyzed by a Shimadzu (TGA-50H) thermal analyzer.

The dielectric relaxation processes of the synthesized polymeric sheets were investigated using a high-resolution impedance analyzer spectrometer (Schlumberger Solartron 1260), supplied with a measuring cell and an electrometer amplifier. Silver conductive paste was used as an ohmic electrode. The complex dielectric permittivity ε*(*ω*) of the polymeric sheet samples can be obtained as follows [14]:

(1) $$\varepsilon^{*}\left(\omega \right)=\varepsilon_{1}-i\varepsilon_{2}=\frac{C\;d}{\varepsilon_{0}U}-i\frac{d}{\omega Z\varepsilon_{0}U}\;$$

where $\varepsilon_{1}\;$and $\varepsilon_{2}$ are the real and imaginary parts of the complex permittivity, respectively. *d* and *U* are the thickness and the cross-sectional area of polymeric sheet samples, respectively. ω is the angular frequency (=2π*f*) and $\varepsilon_{0}$ is the permittivity of vacuum.

## 3. Results and Discussion

### 3.1. Thermogravimetric Analysis

**Figure 2:**
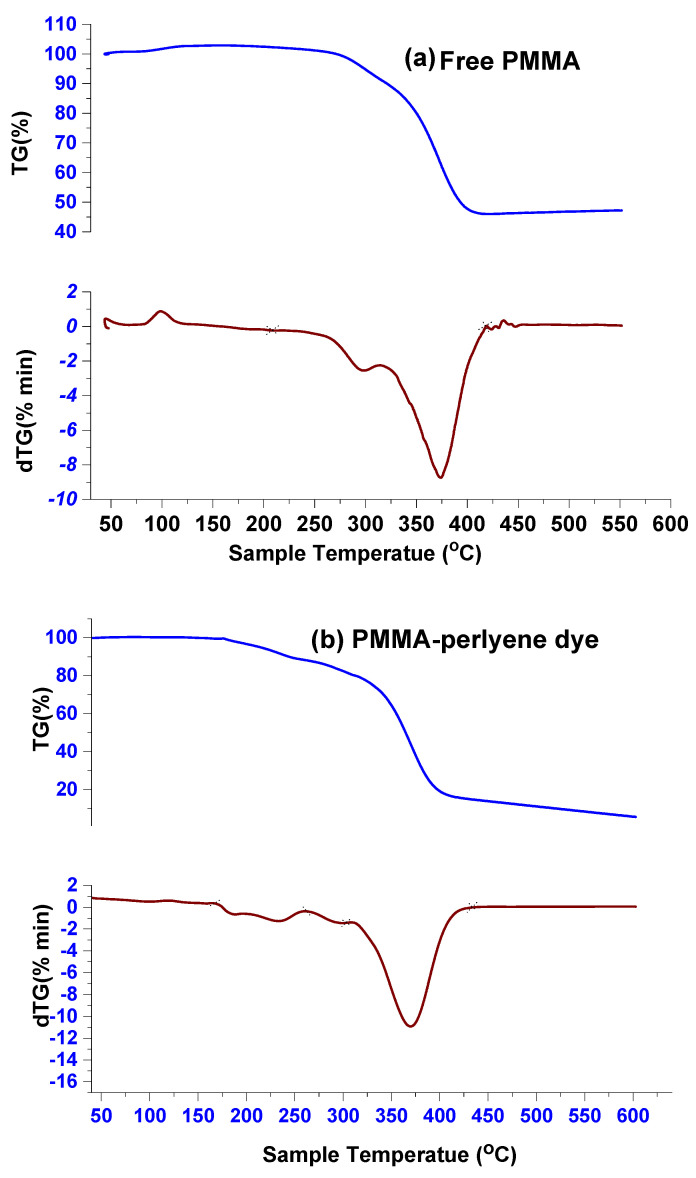

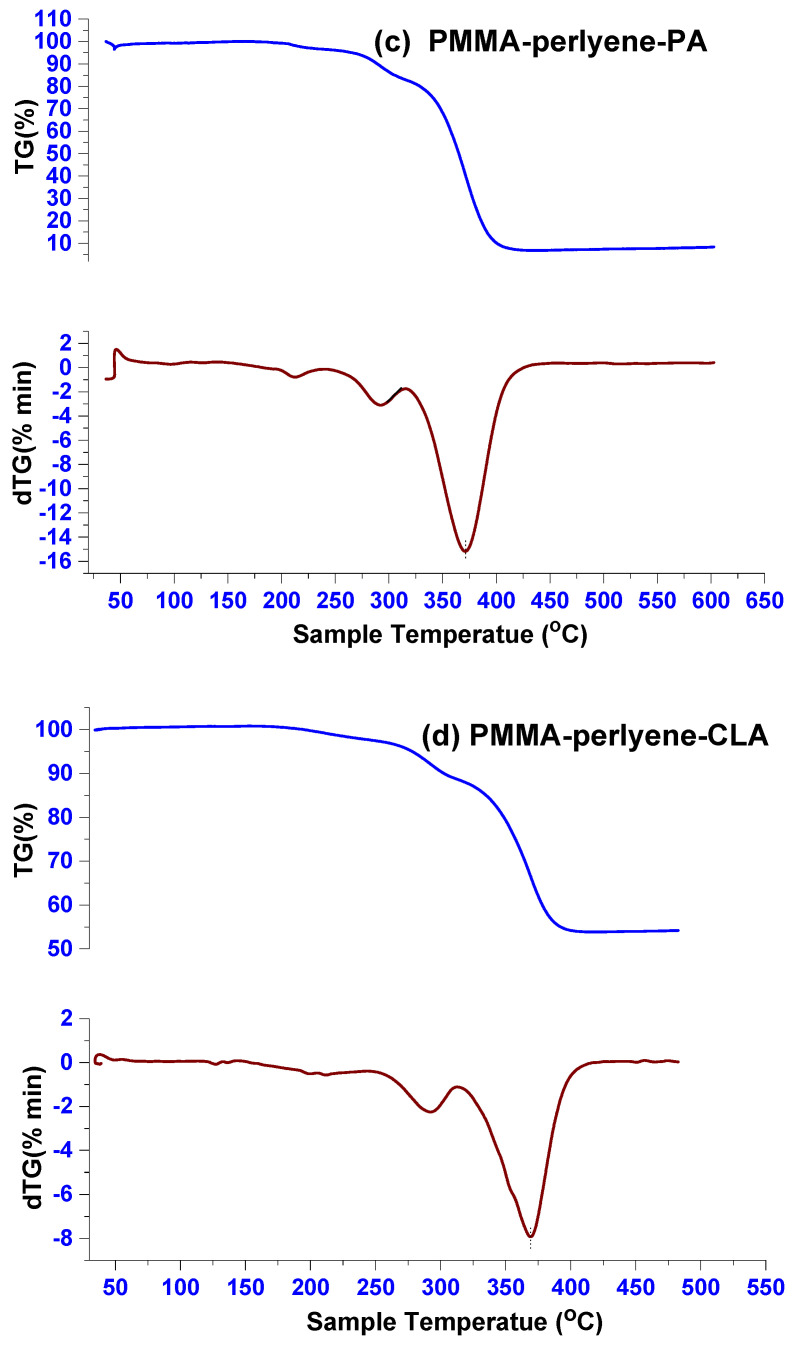
(**a**) TG and dTG curves of free PMMA polymer sheet; (**b**) TG and dTG curves of PMMA-perylene polymer sheet; (**c**) TG and dTG curves of PMMA-perylene-PA polymer sheet; (**d**) TG and dTG curves of PMMA-perylene-CLA polymer sheet.

Figure 2a–d illustrate the degradation of free PMMA, PMMA-perylene dye, PMMA-perylene-PA, and PMMA-perylene-CLA polymeric sheets during thermal decomposition. The obtained data from the TG method show that one to three different stages of fragments. The PMMA polymer sheet (Figure 2a) has only an intense broad band within the temperature range 252–419 °C, which is supported by the maximum dTG peak at 374 °C, accompanied by additional weight loss of 52.65% that can be attributed to the evolution of the carbon dioxide gaseous phase, and few unoxidized carbon atoms are formed as a residual product. In the case of polymer sheets consisting of perylene dye in in situ PMMA (Figure 2b), the first thermal decomposition step is assigned to the decomposition of perylene dye, attributed to the small endothermic dTG at 231 °C and weight loss (found = 10.811%) detectable in the 167–261 °C temperature range. The second thermal degradation stage is due to the decomposition of PMMA, which, displayed in the broad endothermic peak with experimental mass loss, is 82.465% in the 310–429 °C temperature range with dTG peaks at 370 °C. The residual mass is equal to 6.724%, which is attributed to the unoxidized few carbon atoms. Regarding the thermal decomposition of the two PMMA-perylene-PA and PMMA-perylene-CLA polymer sheets (Figure 2c,d), they have three stages at first (193–240 °C), second (243–315 °C), and third (317–435 °C) and first (114–134 °C), second (248–318 °C), and 3rd (319–419 °C) with the dTG peaks at 212, 291, and 371 °C and 121, 294, and 374 °C, respectively. The thermal decomposition steps in the N_2_ environment pass through three species as donor (perylene dye), acceptor (PA or CLA), and PMMA decompositions and the residual product is un-oxidized free carbon atoms. These thermal decomposition steps have a weight loss that changes with various thermodynamic behaviors [18]. A substantially broad endothermic peak with a mass loss shown at the temperature range of 317–435 °C to be 88.183% and 43.377% for PMMA-perylene-PA, and PMMA-perylene-CLA polymer sheets, respectively, can be attributed to the acceptors and PMMA species and residual unoxidized carbon atom formation. The observed difference between mass loss curves of PMMA-perylene-PA and PMMA-perylene-CLA polymer sheets is related to the difference in the molecular formula of acceptor that the PA acceptor has a rich amount of oxygen atoms allowed to facilitate the oxidizing of free carbon atoms. The thermal analysis of the PMMA-perylene-iodine polymer sheet was not performed because the polymer sheet contains iodine, as iodine has sublimation properties, which does not give accurate thermal cracking values.

### 3.2. Kinetic Thermodynamic Study

To determine the kinetic thermodynamic parameters dependent on the thermogravimetric curves regarding the non-isothermal decomposition reactions, there are several equations [19,20,21,22,23,24,25,26] that can be used to analyze a TGA curve. Concerning the estimation of thermodynamic parameters of our synthetic polymeric sheets of PMMA-perylene dye, PMMA-perylene-PA, and PMMA-perylene-CLA, Table 1 contains some parameters such as range of stability, dTG peaks, and kinetic thermodynamic parameters. The Coats–Redfern relationship [21,22] is employed to calculate the kinetic thermodynamic parameters (ΔH, ΔS and ΔG). The results of the thermodynamic data of the thermal decomposition peak in each of three materials I, II, and III are calculated based on the Coats–Redfern relationship [21,22] as follows.

(2) $$\ln\left(\frac{-\ln\left(1-\alpha \right)}{T^{2}}\right)=\ln\left(\frac{ZR}{\varphi E}\right)-\frac{E}{RT}\;$$

where α and φ are the fraction of the fraction decomposed at time t and the linear heating rate, respectively. *R* is the gas constant and *E* is the activation energy in kJ mol^−1^.

A plot of $\ln\left(\frac{-\ln\left(1-\alpha \right)}{T^{2}}\right)$ against $\frac{1}{T}$ was found to be linear, from the slope of which E was calculated and *Z* (Arrhenius constant) can be deduced from the intercept. The enthalpy of activation ΔH and the free energy of activation, ΔG, can be calculated (Table 1) via the equation

(3) $$\Delta H=E-RT_{m};\;\Delta G=\Delta H-T_{m}\Delta S$$

The negative values of activation entropy (−Δ*S*) revealed that the associated charge transfer compounds in in situ polymeric sheets are more ordered than the initial reactants; so, the thermal degradation steps of charge transfer compound are non-spontaneous and thermally stable. From the Arrhenius plots, it was found that the correlation coefficient factors of the thermal degradation stages are in the range of ~0.99, showing a good linear fit.

### 3.3. Dielectric Polarization and Relaxation Processes

**Figure 3:**
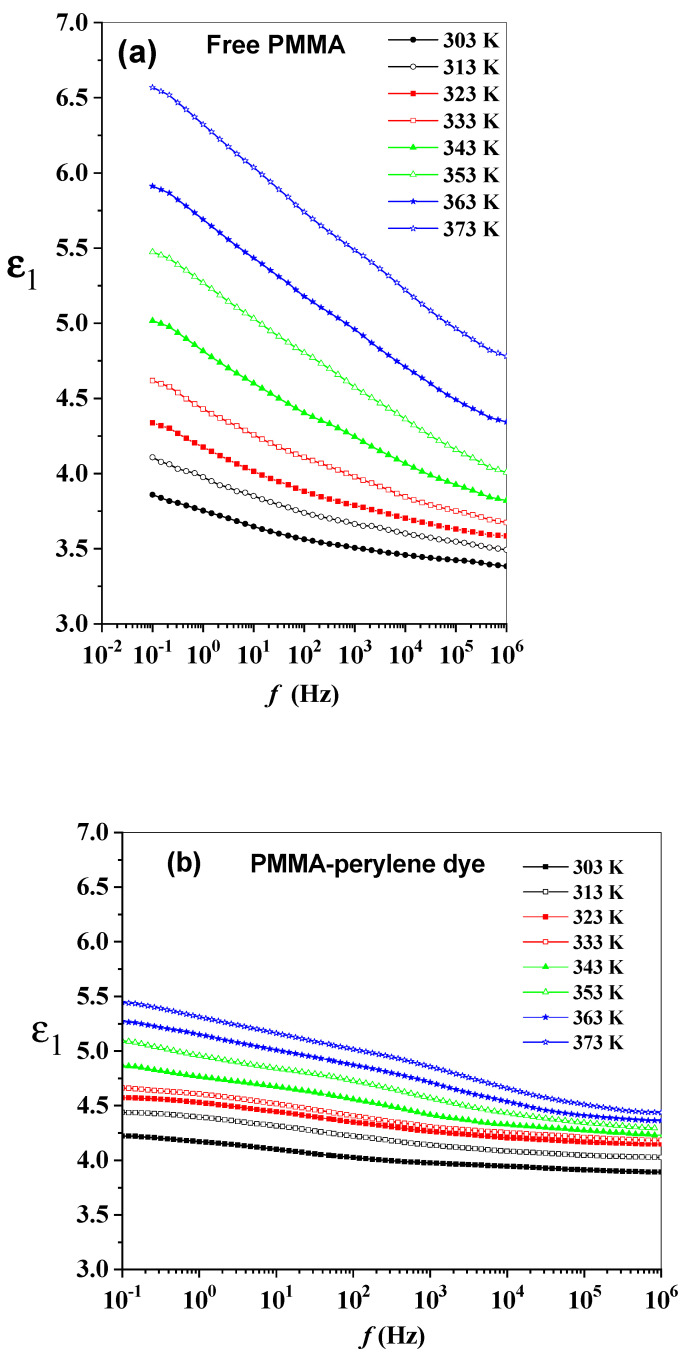

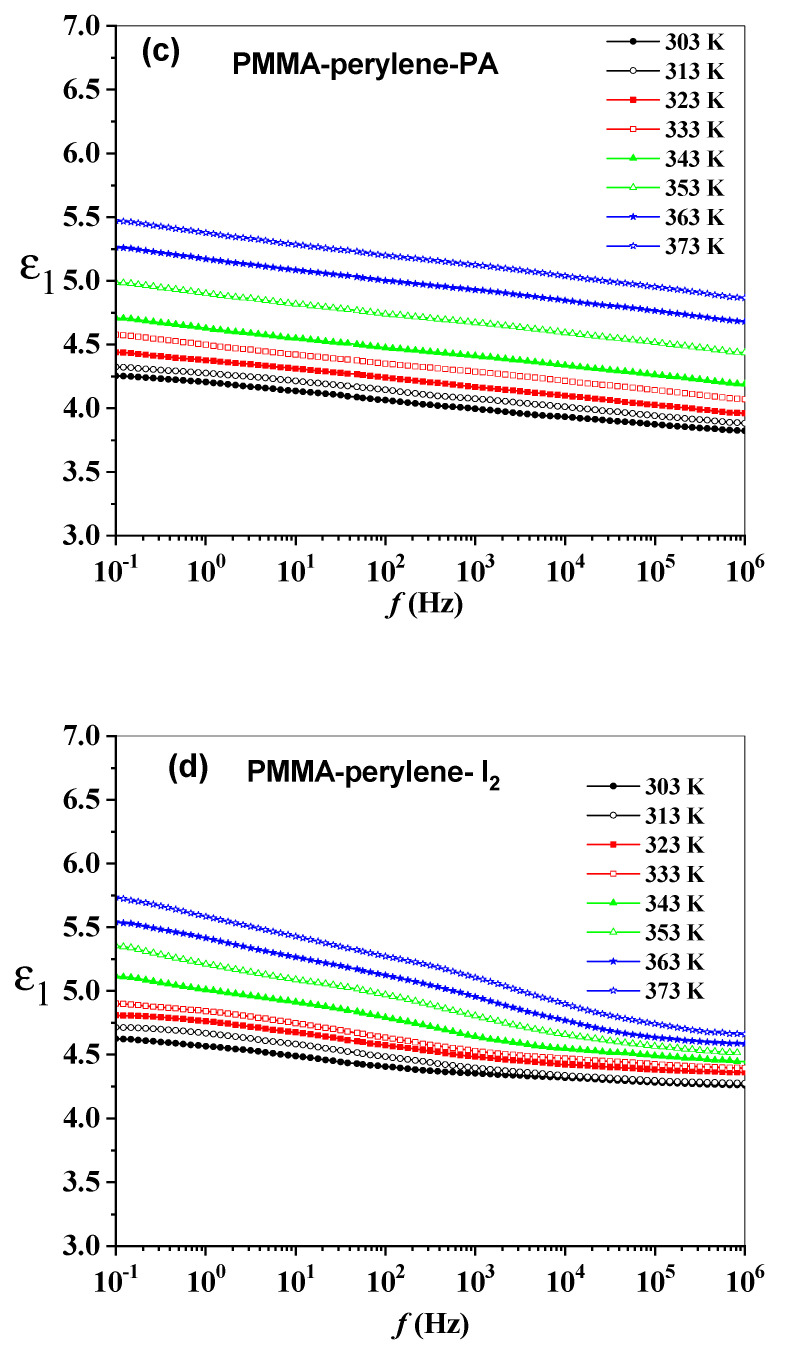

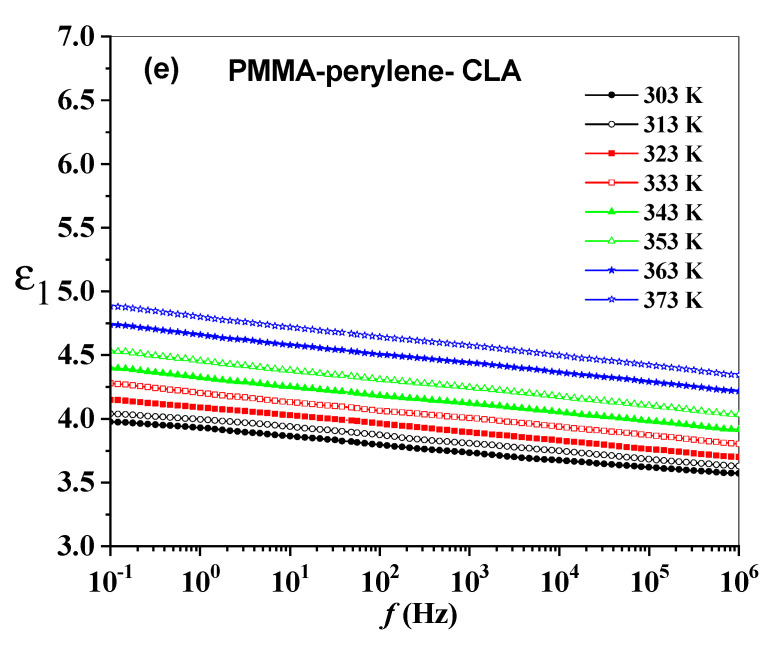
(**a**–**e**): The frequency dependence of the ε1 of the synthesized polymeric sheets.

Electric permittivity$\;\varepsilon^{*}\left(\omega \right)$ is the furthermost meaningful and common demonstration of the dielectric data in polymers. The energy storage ability of the polymer can be shown by the real part of the dielectric permittivity ($\varepsilon_{1}$). Alternatively, the energy dissipation (absorption of the polymer) due entirely to the material medium can be shown by the imaginary component ($\varepsilon_{2}$). For the synthesized polymeric sheets, the frequency dependences of $\varepsilon_{1}$ and $\varepsilon_{2}$ were obtained at a temperature range of 303–373 K and over a frequency ranging between 0.1 Hz and 1 MHz. The frequency and temperature dependences of $\varepsilon_{1}$ for the polymeric sheets are shown in Figure 3a–e. An increase in$\;\varepsilon_{1\;}$with decreasing frequency over the investigated temperature range (dispersion of$\;\varepsilon_{1}$) was observed. Alternatively, at constant frequency, a decrease in$\;\varepsilon_{1\;}$with decreasing temperature (the mobility of polar molecules increases) was observed.

**Figure 4:**
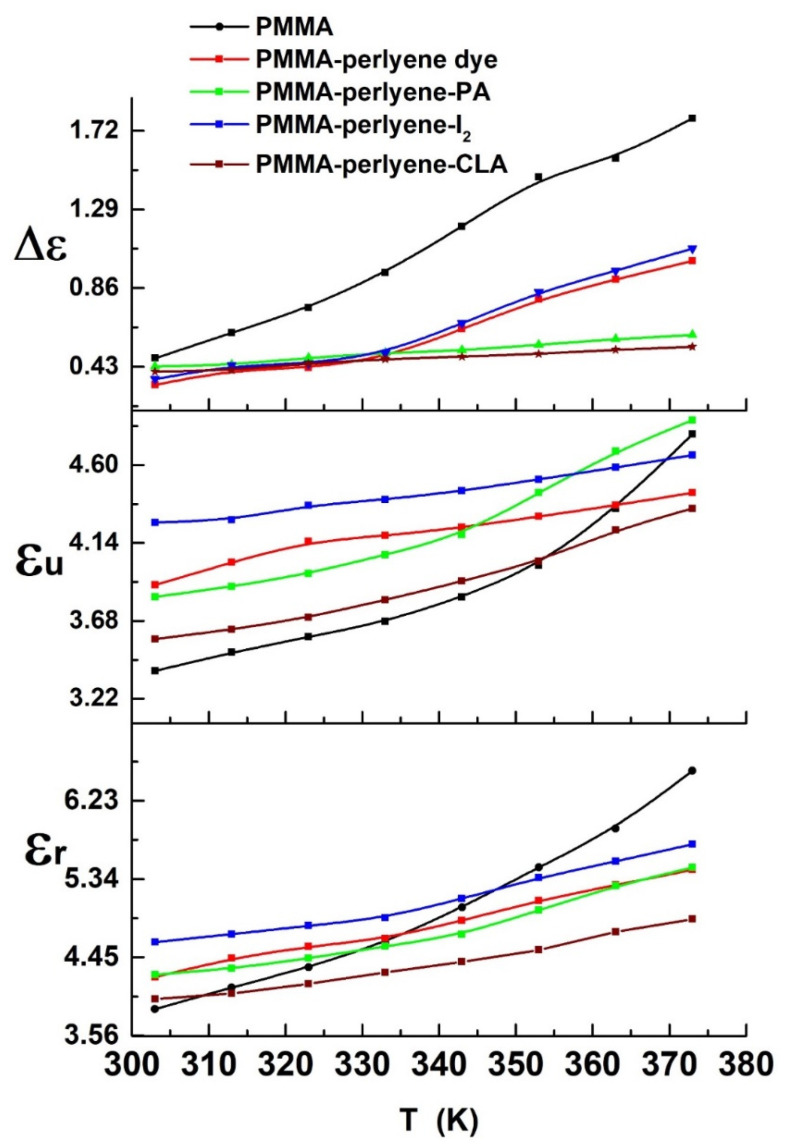
The temperature dependence of $\varepsilon_{r}$, $\varepsilon_{u}\;and\;\Delta \varepsilon \;$of the synthesized polymeric sheets.

The baseline of $\;\varepsilon_{1\;}$without the contribution of free charge migration and dipole orientation can be obtained from unrelaxed $\;\varepsilon_{1\;}$ ($\varepsilon_{u}\;or\;\varepsilon_{\infty };\;\text{i.e.,}\;\varepsilon_{1\;}\;measured\;at\;f\to \infty )$, which is identified as the plateau reached in $\;\varepsilon_{1\;}$ at high frequencies when all polarization fluctuations due to orientation polarization cease. The decrease in $\;\varepsilon_{1\;}$with increasing frequency can be described based on several types of polarization (orientation, and electronic). All types of polarizations may be effective in low frequencies. However, at higher frequencies, the $\;\varepsilon_{1\;}$ may be quite small because some polarization types may not be effective. Temperature dependency and the contribution of the different types of polarizations may not be same. Additionally, the higher degree of dipole orientation in the polymeric sheets can be obtained from the relaxed permittivity or static permittivity ($\varepsilon_{r}\;or\;\varepsilon_{s};\;\text{i.e.,}\;\varepsilon_{1\;}\;measured\;at\;f\to 0).$ The oscillator or dielectric strength (Δε) of the polymeric sheets can be describes from the relation, $\Delta \varepsilon =\varepsilon_{r}-\varepsilon_{u}$. The dielectric strength of the polymer provides information about the voltage that the synthesized polymeric sheet can withstand before breakdown occurs [27]. In addition, the dielectric strength of the synthesized polymeric sheet represents the effective moment of the orienting unit increases with a temperature increase on the whole, in which certain units of the chain are gradually motivated with increasing temperature, and the dielectric polarization is enhanced. The dielectric relaxation time is gradually decreased with increasing temperature. This is evidence that the response time of the polymeric chain polarization is progressively harmonious with the alternative electric field [28]. The temperature dependence of $\varepsilon_{r}$, $\varepsilon_{u}\;and\;\Delta \varepsilon \;$of the synthesized polymeric sheets are shown in Figure 4. At higher frequencies, electric dipoles are unable to follow the fast variations in the AC field, resulting in low values of $\varepsilon_{r}$. At low frequencies, higher values of $\varepsilon_{r}$ were observed as a result of the electric dipoles responding faster with the AC field. In addition, space charge polarization occurred due to presence of different boundary regions and a difference in conductivity, so dielectric constant increases with the increment of temperature [29,30,31]. Moreover, at 303 K, Figure 4 shows that the $\varepsilon_{u}$ has a value of 3.42, 3.91, 3.87, 4.28 and 3.62; $\varepsilon_{r}$ has a value of 3.86, 4.22, 4.25, 4.62 and 3.97; and the Δε has a value of 0.44, 0.31, 0.38, 0.34 and 0.35 for free PMMA, PMMA-perylene dye, PMMA-perylene-PA, and PMMA-perylene-CLA, respectively.

**Figure 5:**
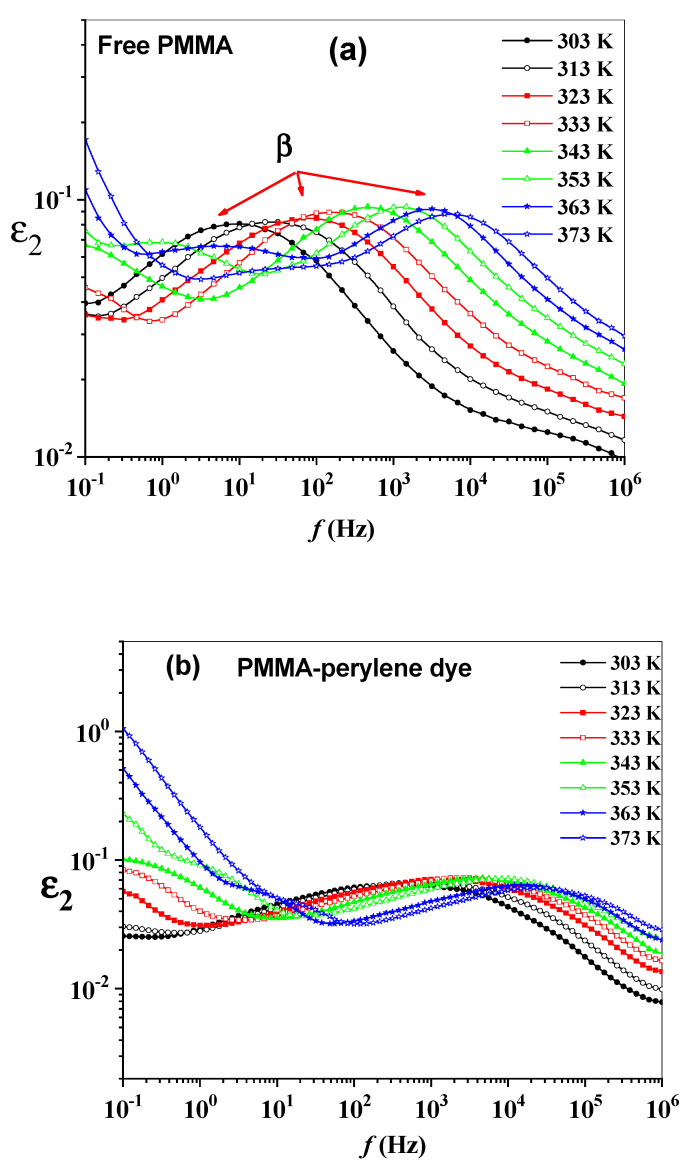

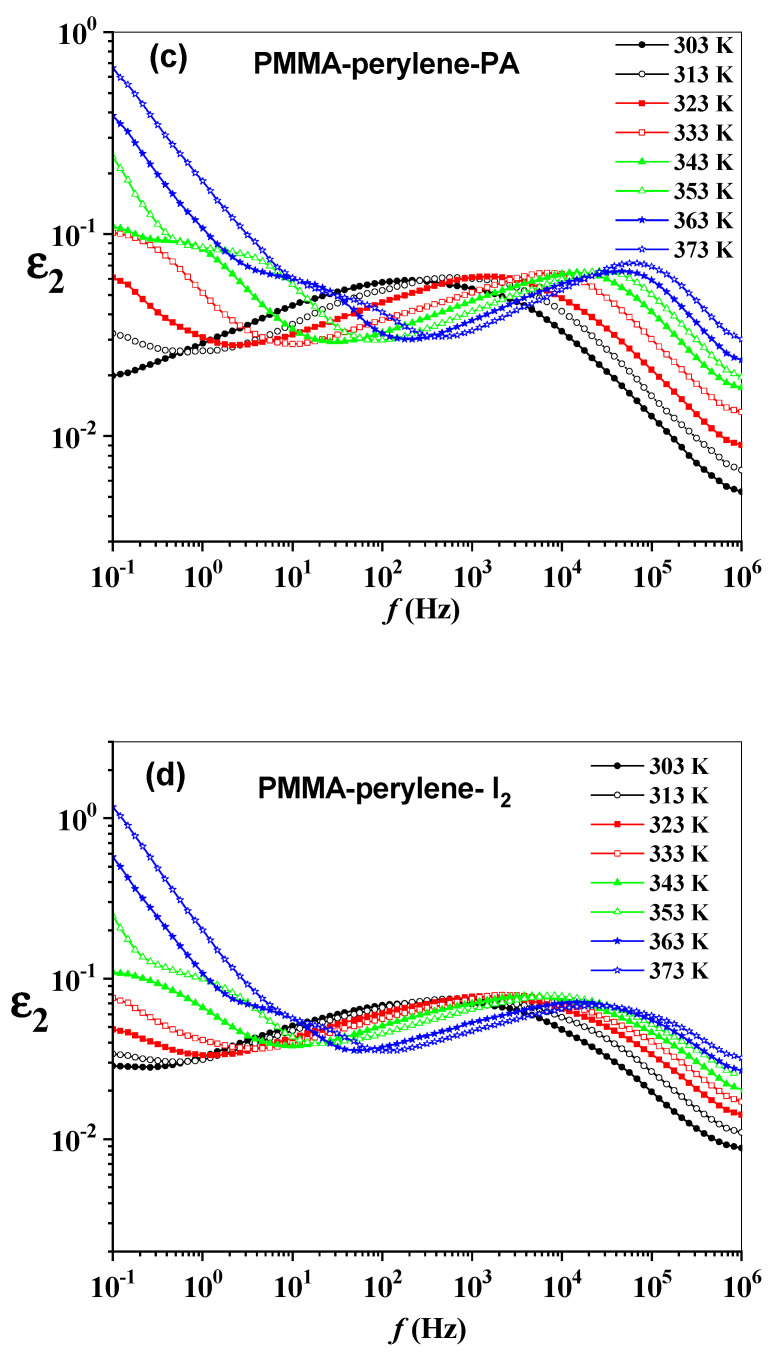

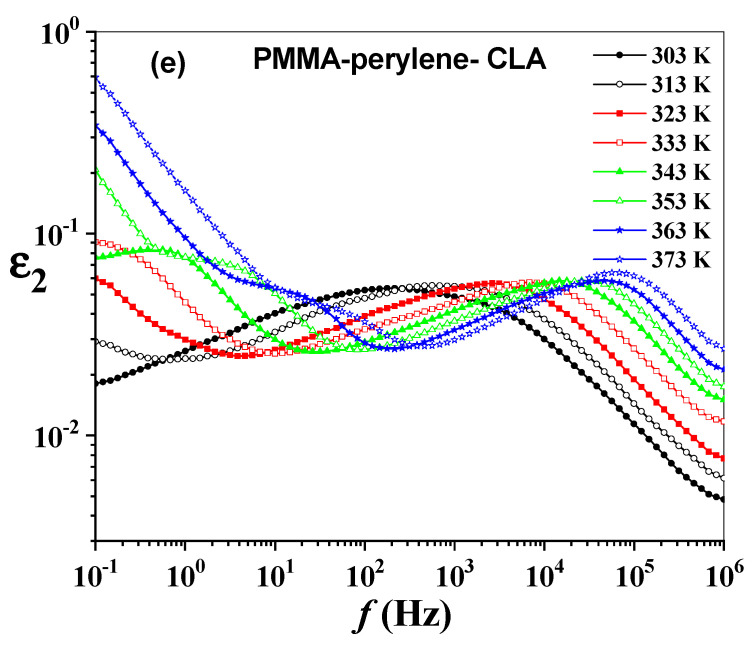
(**a**–**e**): The frequency dependence of the $\varepsilon_{2}$ of the synthesized polymeric sheets.

In general, dielectric losses exhibit a bell-shaped curve. The dielectric loss peaks for most polymers are asymmetric (distorted) and broad. The frequency dependence of $\varepsilon_{2}$ at different temperatures for the polymeric sheets are shown in Figure 5a–e. For temperatures below 353 K, a broad second dielectric relaxation peak appears in ($\varepsilon_{2}$) for all polymeric sheets corresponding to the *β* relaxation. The magnitude of the maximum height of the *β* peaks are temperature independent. The intra molecular fluctuations may be the source of *β* relaxation that is affected by partial rotation of the side group around the bond, linking it to the main chain (heterogeneous local environment) [32,33]. For temperatures above 353 K, two peaks of dielectric relaxation appear in $\varepsilon_{2}$ for all polymeric sheets corresponding to the *α* and *β* relaxation. *α* relaxation is associated to the glass transition of the polymer and refers to cooperative transitions.

The experimental relaxation behavior of the above results can be described by the empirical dispersion function reported by Havriliak–Negami (HN) [34,35].

(4) $$\varepsilon^{*}\;\left(\omega \right)=\varepsilon_{u}+\frac{\varepsilon_{r}-\varepsilon_{u}}{{\left[1+{\left(i\omega \tau \right)}^{\alpha }\right]}^{\beta }}\;\;\;\;with\;\;\;0\leq \alpha ,\beta \leq 1$$

Here, α and β are exponent parameters ranging between 0 and 1. For the Debye dispersion function, α = 1, β=1, Cole–Cole dispersion β = 1, α≠1, whereas for the Cole–Davidson function, α = 1, β≠1.

**Figure 6:**
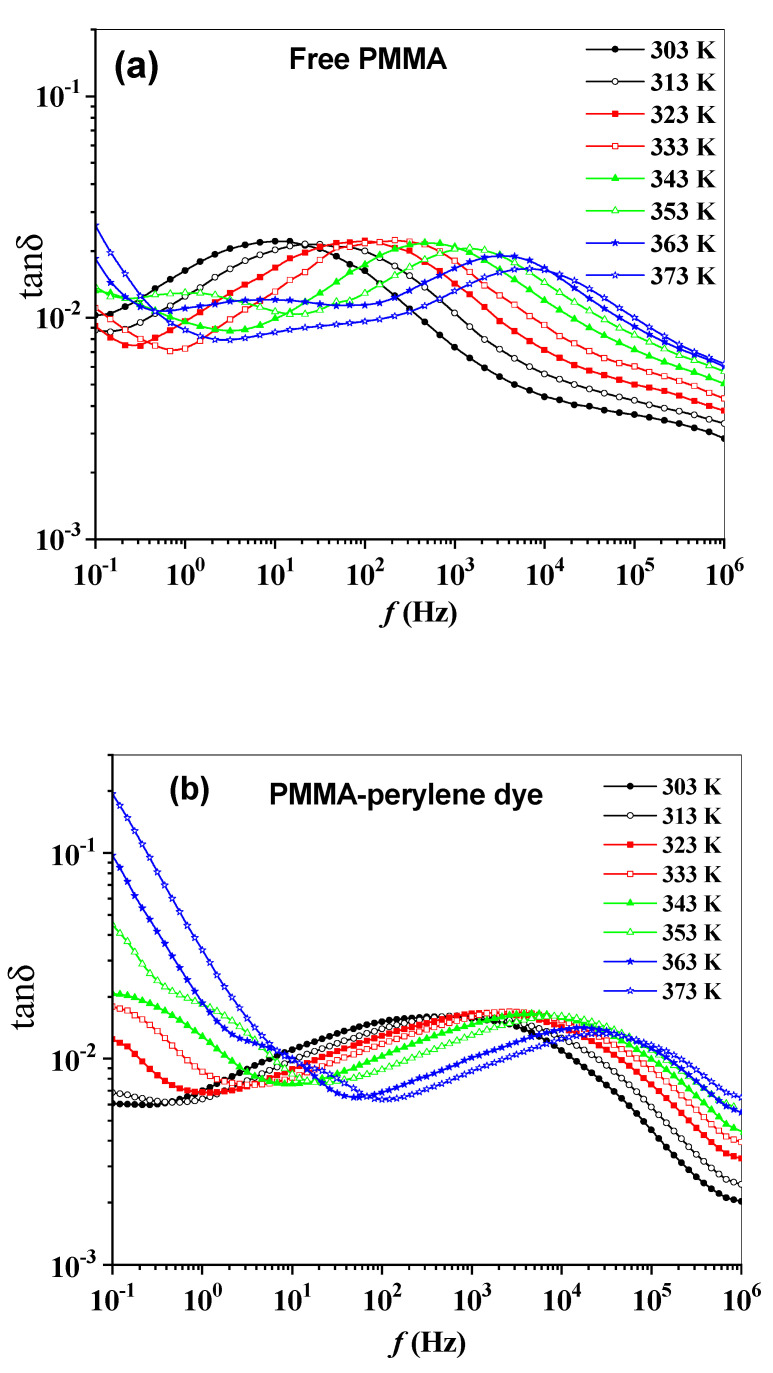

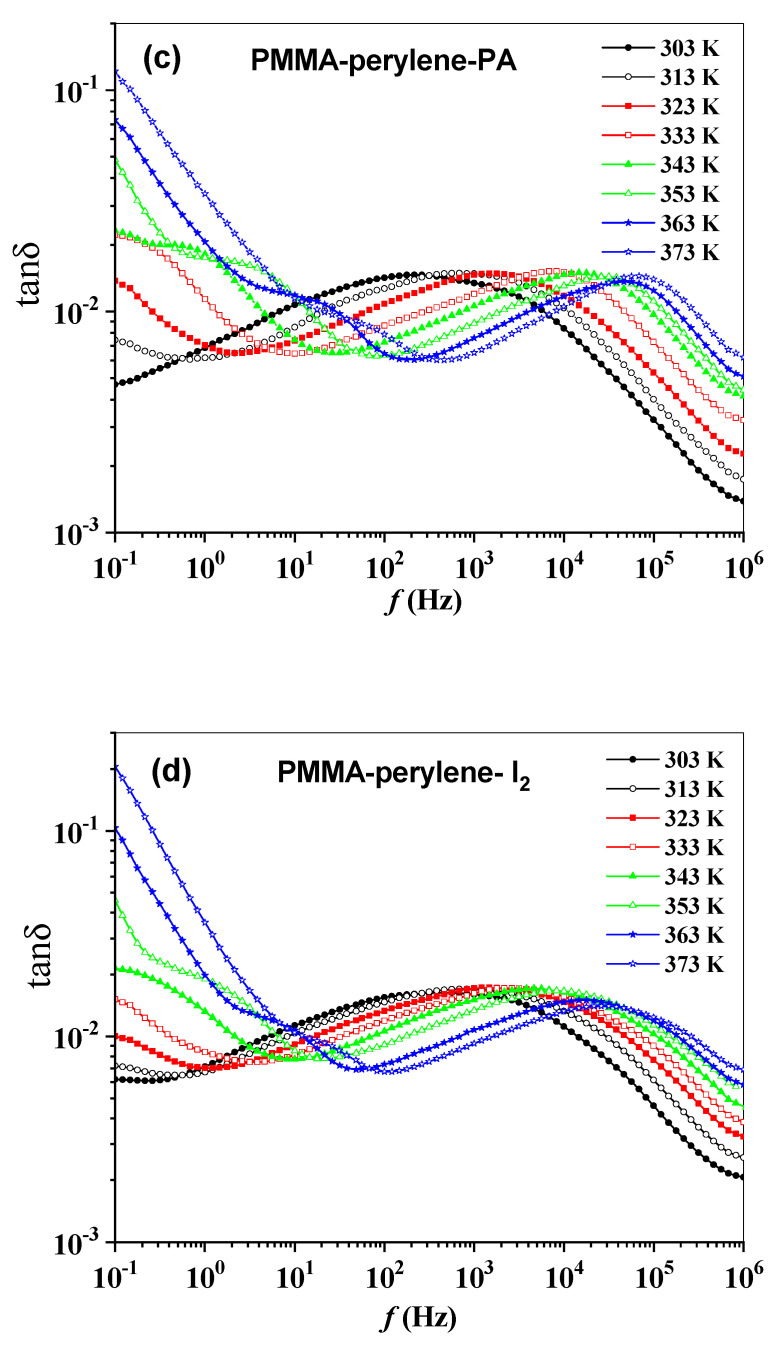

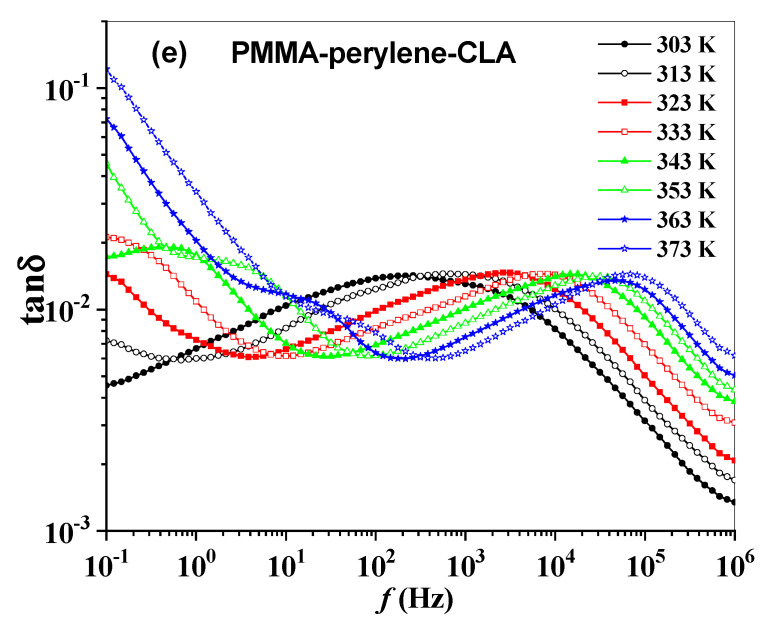
(**a**–**e**): The frequency dependence of the tan δ of the synthesized polymeric sheets.

For many electrical engineering applications, the loss tangent or so-called dissipation factor$\;(tan\;\delta =\varepsilon_{2}/\varepsilon_{1}$) is also frequently used to describe the amount of energy dissipated inside the polymer due to the alternative field. It is an amount of polymer polarization inertia with respect to the applied electric stimulus. The *tan δ* can be used to demonstrate a dielectric of polymer in cases where the geometry of the polymer is not known. The frequency and temperature dependences of tan δ for the polymeric sheets are shown in Figure 6a–e. The loss tangent at lower frequencies is related to the loss of the interfacial polarization, while the loss tangent at higher frequencies is related to the change in dipole-relaxational polarization [36].

Another representation of the complex relative permittivity ($\varepsilon^{*}$) is the complex electric modulus ($M^{*}$). The $M^{*}$ formalism gives a clearer characterization of the dipolar relaxation mechanisms of the synthesized polymeric sheets. The $M^{*}$ formalism is described by the following relation [25].

(5) $$M^{*}=\frac{1}{\varepsilon^{*}}==M_{1}+iM_{2}=\frac{\varepsilon_{1}}{\varepsilon_{1}^{2}+\varepsilon_{2}^{2\;}}+i\;\frac{\varepsilon_{2}}{\varepsilon_{1}^{2}+\varepsilon_{2}^{2\;}}$$

**Figure 7:**
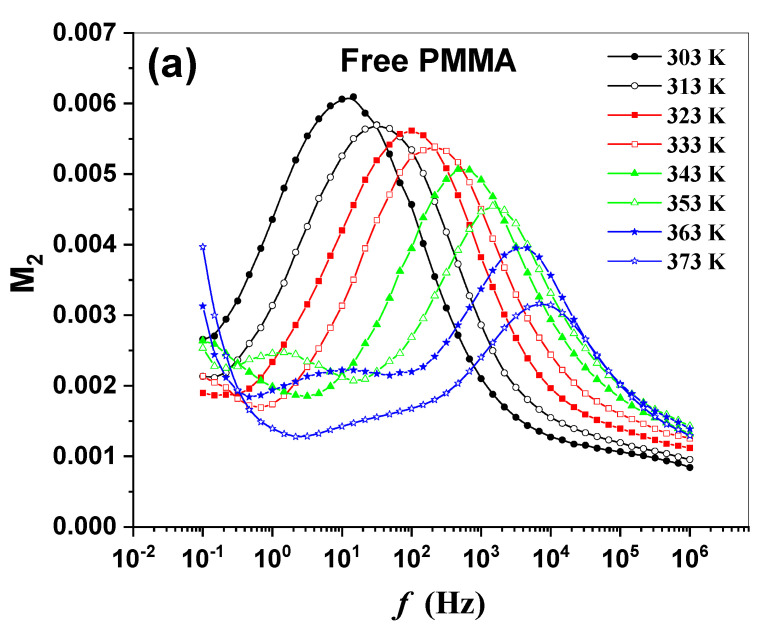

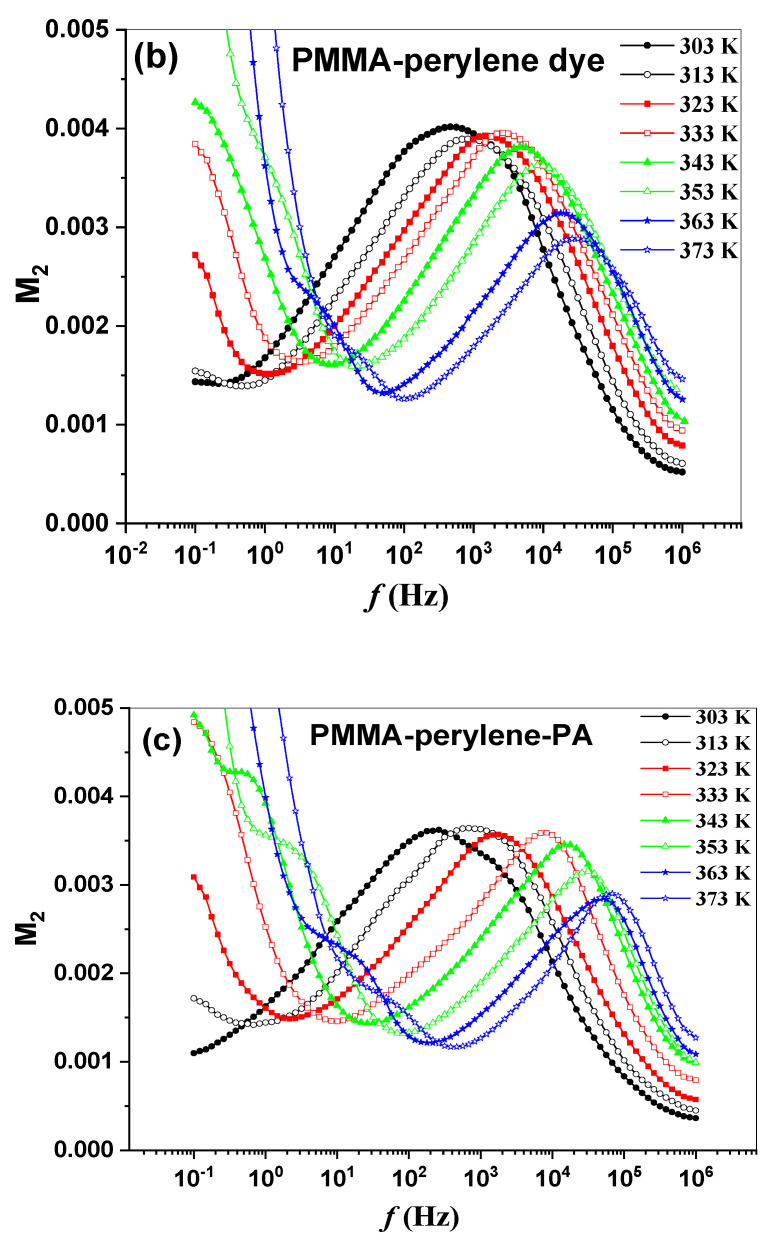

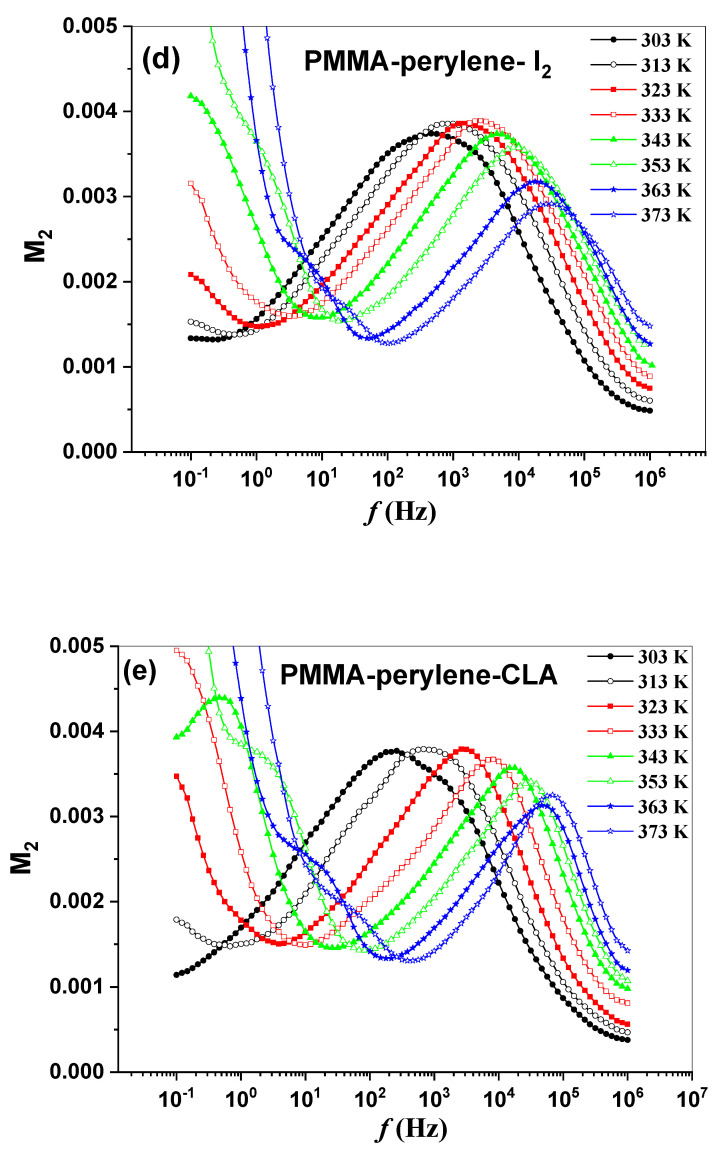
(**a**–**e**): The frequency dependence of the $M_{2}$ of the synthesized polymeric sheets.

The frequency and temperature dependences of the imaginary part of the electric modulus ($M_{2}$) for the polymeric sheets are shown in Figure 7a–e. At low frequencies, large contributions of nonlocal relaxations of the polymeric sheets can be successfully suppressed by using $M_{2}$ plots.

The characteristic relaxation time for the β-relaxation peak in the synthesized polymeric sheet has been defined as the reciprocal frequency of the peak maximum ($\tau_{max}=1/f_{max}$) where $f_{max}$ is the peak frequency of the $\varepsilon_{2},M_{2}\;and\;tan\delta$. The relaxation process in the synthesized polymeric sheets networks can be determined via the Arrhenius equation [37,38]:

(6) $$f_{max}=f_{0}\exp\left(-\frac{\Delta E_{a}}{kT}\right)$$

where$\;f_{0}$ $=f_{max}$ at T→0, and k is the Boltzmann constant. The transition probabilities between consecutive minimum energy configurations of the dipoles (minima of the potential valleys) are related with the activation energy$\;\Delta E_{a}$. In this case, as follows from Equation (6), with increasing temperature, the relaxation time decreases exponentially, and the activation energy does not show any dependence on temperature. The values of the relaxation activation energy parameter ($\Delta E_{a}$) for the synthesized polymeric sheets are compared in Table 2. This table shows that the values of the activation energies ($\Delta E_{a}$) for the molecular rotation in the perylene complexes of (iodine, picric acid, and chloranilic) fall in the range of 0.51–0.79 eV. These values of the activation energies ($\Delta E_{a}$) are comparable with those for the less hindered rotation in free PMMA. Higher activation energies for the C3 rotation were observed for the charge transfer complexes, especially the strong complexes of perylene with nitro (picric acid) and halogens (chloranilic acid). The higher-order axis of rotation (i.e., C3) is called the major axis of rotation. When the major Cn axis has even n, it has a C2 process bound to that axis. The C2 vertical axes and their associated processes must be indicated by initial and double uppercase letters. Ang and Dunell [39] reported a connection between $\Delta E_{a}$ for the C3 rotation to predictable structural variation in perylene dye as a result of charge transfer to an electron acceptor [39]. Their efforts were not promising owing to a shortage of accurate molecular structural data for many of the perylene dye complexes. $\Delta E_{a}$ for the C3 rotation increases with an increase in the electron affinity of the acceptor in the series of PA > CLA > I_2_. Since electron affinity is a measure of the strength of the acceptor type, the rotational barrier in the donor dye (perylene) increases as the acceptor strength increases. As for the molecular rotation, the $\Delta E_{a}$ of the complexes with PA and CLA is higher than that of iodine. In perylene-I_2_, $\Delta E_{a}$ for the rotation of iodine molecule about the σ-π* bond is equivalent to that for the aromatic character of perylene reorientation. We can conclude that perylene and perylene-I_2_ show similar dynamical behavior [40,41,42,43].

### 3.4. AC Electrical Conductivity Dispersion

**Figure 8:**
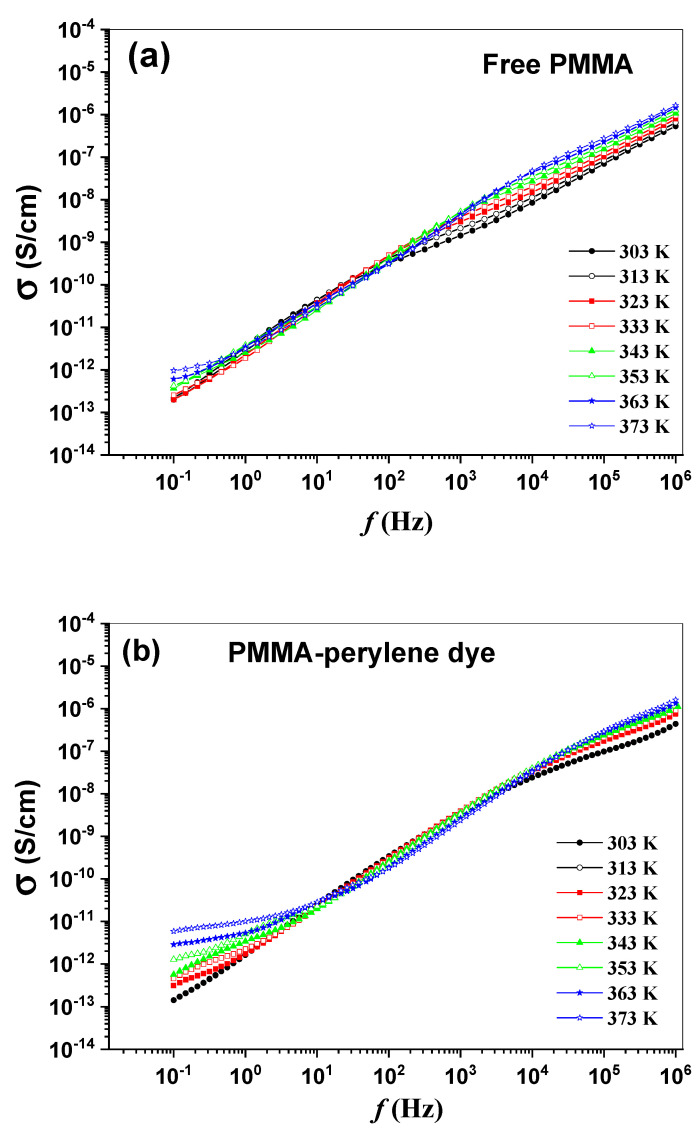

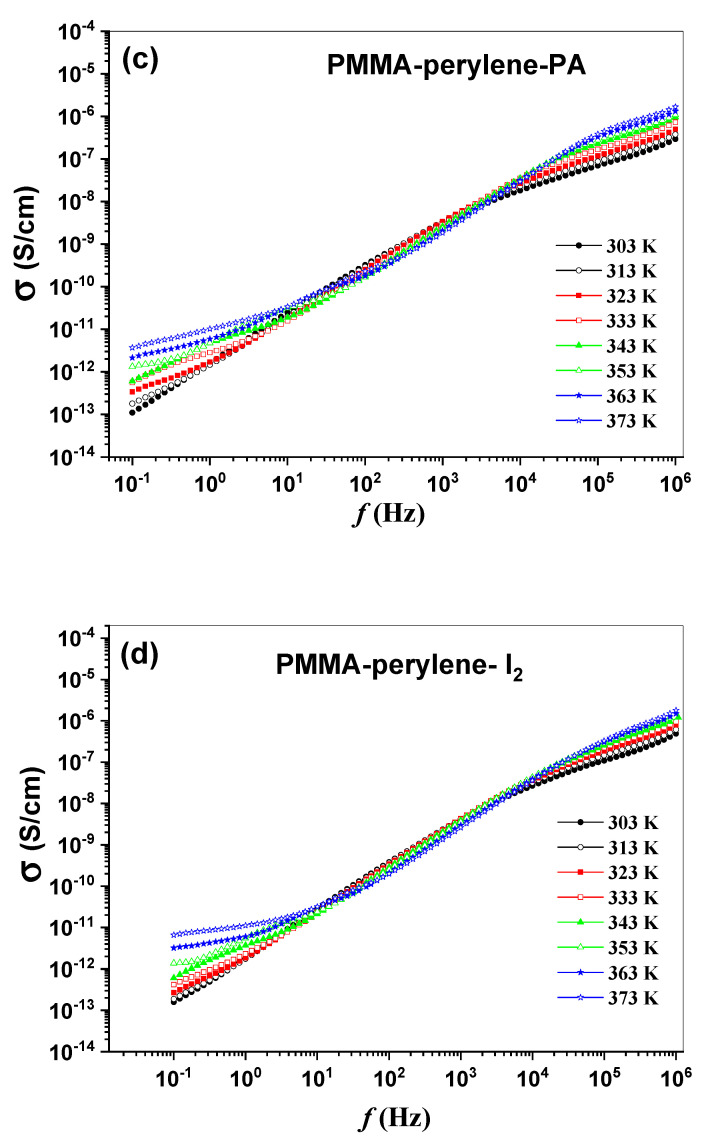

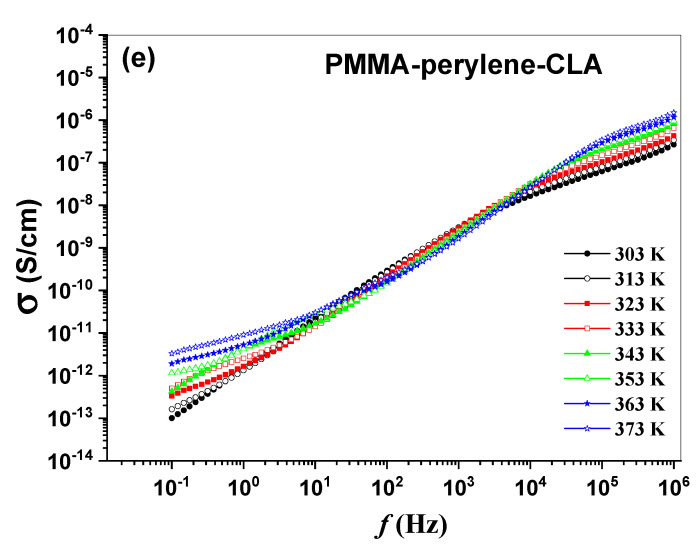
(**a**–**e**): The frequency dependence of the σ (ω) of the synthesized polymeric sheets.

Figure 8a–e show the real part of the complex electrical conductivity σ(ω) spectra over the temperature range 303–373 K and frequency range 0.1 Hz–1 MHz for the synthesized polymeric sheets. These figures are characterized by the transition above threshold angular frequency (*ω*_0_) from a low-frequency direct electrical conductivity ($\sigma_{dc}$) plateau to a dispersive high-frequency range. The dc conduction in the disordered polymer appears to be associated to the presence of a polarization process, which is associated with a short range of mobile electric charges. These mobile electric charges contribute to the $\sigma_{dc}\;$at low frequency region [44]. After the critical angular frequency, the σ(ω) dispersion of the polymeric sheets shows two distinct regions with increasing frequency. The dependence of the σ(ω) on the angular frequency is found to follow the form:

(7) $$\sigma \left(\omega \right)=\sigma_{dc}+A^{’}\omega^{S_{1}}+B\omega^{S_{2}}\;\;$$

where $A^{\prime }$ and *B* are two constants, while *s*_1_ and *s*_2_ are fractional exponents. At the first region, the σ(ω) is nearly temperature independent where $S_{1}\approx 1$, demonstrating obviously that the nearly constant loss (NCL) phenomenon is predominant. In this model, there is a negligible frequency dependence of $\varepsilon_{2}$ [44,45]. Polymer/backbone segments are greatly flexible, and they respond with frequency variation due to rapid changes in the polarity of the applied field, as well as in temperature. The origin of NCL to the polymer chain relaxation strongly depends upon temperature for the pure polymeric system. However, Das et al. reported that NCL has been attributed to the nature of polymer relaxation rate for polymer salt concentration [46]. According to studies that have been reported so far, the following properties are recognized for the NCL. The dielectric loss does not depend on frequency so much and follows such a power law as ($\varepsilon_{2}\sim f^{-\nu },\;\nu =0.1–0.3$) [47]. In the second region, $S_{2}\approx 0.6$ and independent of temperature may be attributed to the quantum mechanical tunneling mechanism of charge carriers over the potential barrier separating two energetically favorable centers in a random distribution. The QMT mechanism has been reported to be the dominant operating conduction mechanism in many synthesized polymeric materials [48,49,50,51].

**Figure 9:**
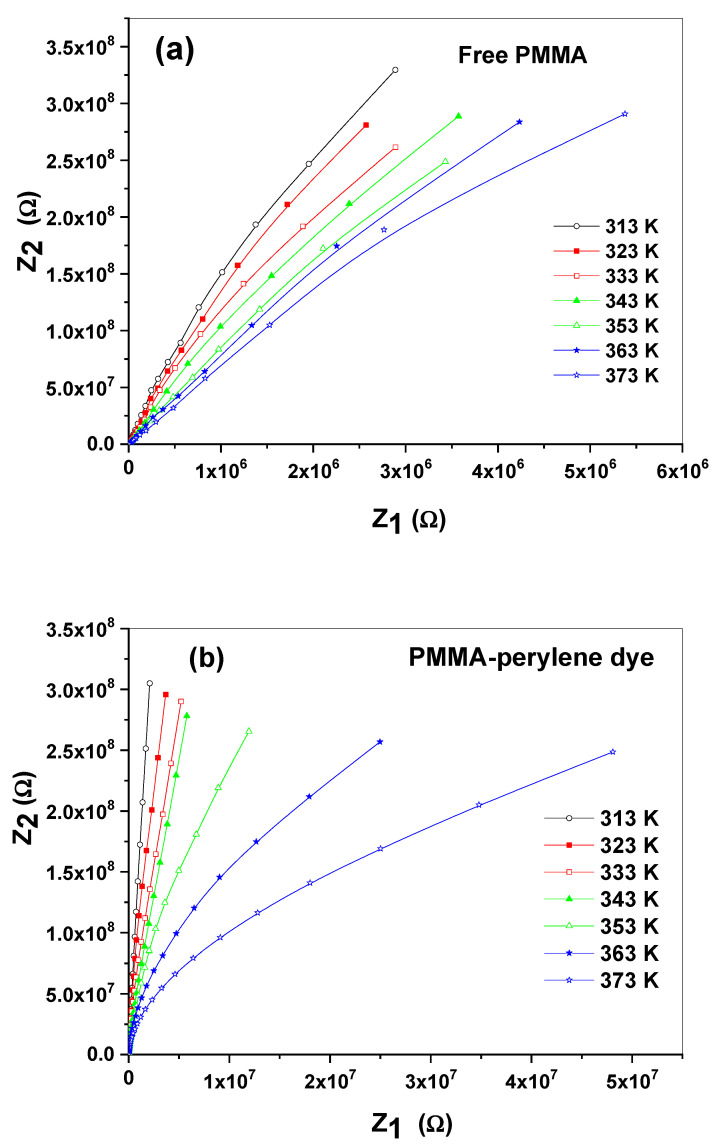

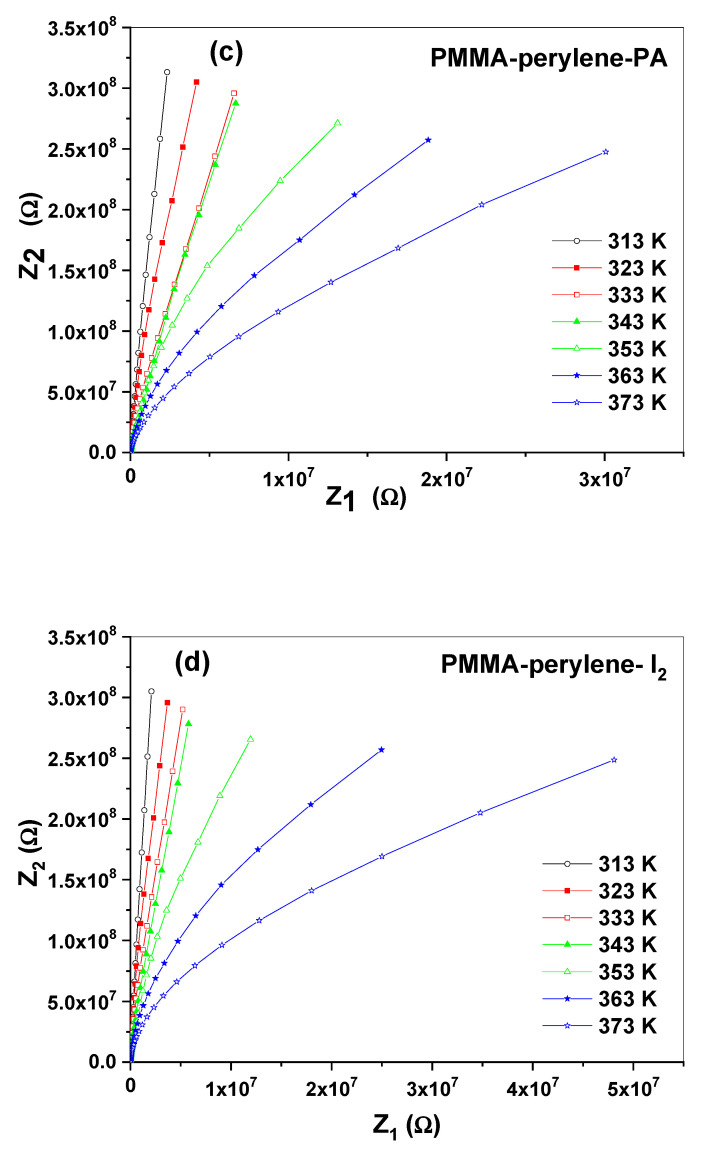

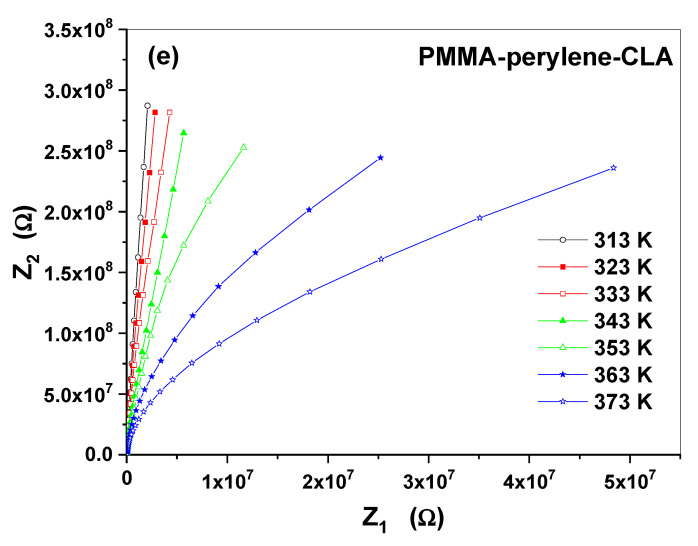
(**a**–**e**): The complex impedance plot of the synthesized polymeric sheets at different temperatures.

The complex impedance ($Z^{*}\left(\omega \right))$ of the polymeric sheets can be analyzed by using the following relation [52]:

(8) $$Z_{1}\;\left(\omega \right)=\frac{G}{\left(G^{2}+\omega^{2}C^{2}\right)}$$

(9) $$Z_{2}\;\left(\omega \right)=\frac{C\omega }{\left(G^{2}+\omega^{2}C^{2}\right)}$$

where *G* is the conductance of the investigated polymeric sheet. Figure 9a–e display the Nyquist plot (complex impedance plot) of the polymeric sheets at different temperatures. The Nyquist plot consists of one semicircle demonstrating the bulk resistance of the polymeric sheets. The semicircle decreases and bends towards the $Z_{1}$ (ω) axis with increasing temperature. The equivalent circuit can be expressed by the capacitor and resistor in parallel (parallel RC element) [53,54].

## 4. Conclusions

Perylene dye and acceptors (iodine, picric acid and chloranilic acid) in an in situ PMMA polymer were synthesized in methanol solvent at room temperature. TG/dTG showed that the synthesized charge transfer compounds in an in situ PMMA polymer are thermally stable. The calculated kinetic and thermodynamic parameters for PMMA-perylene dye are *E* = 99 (kJ mol^−1^), Δ*S* = −82 (J mol^−1^ K^−^^1^), Δ*H* = 96 (J mol^−1^ K^−1^) and Δ*G* = 138 (J mol^−1^ K^−1^), for PMMA-perylene-PA are *E* = 46 (kJ mol^−1^), Δ*S* = −155 (J mol^−1^ K^−1^), Δ*H* = 44 (J mol^−1^ K^−1^) and Δ*G* = 99 (J mol^−1^ K^−1^), and for PMMA-perylene-CLA are *E* = 65 (kJ mol^−1^) Δ*S* = −26 (J mol^−1^ K^−1^), Δ*H* = 54 (J mol^−1^ K^−1^) and Δ*G* = 54 (J mol^−1^ K^−1^). The frequency dependences of the dielectric properties of polymeric sheet samples were obtained over the frequency range 0.1 Hz–1 MHz at temperatures ranging between 303 and 373 K. Two main mechanisms are observed to describe the molecular motions in the synthesized polymeric sheets; at the lower temperature regime, the β-relaxation is dominant while the segmental α relaxation is dominant at higher temperatures. It was found that the $\varepsilon_{u}$ has a value of 3.42, 3.91, 3.87, 4.28 and 3.62; $\varepsilon_{r}$ has a value of 3.86, 4.22, 4.25, 4.62 and 3.97 and Δε has a value of 0.44, 0.31, 0.38, 0.34 and 0.35 for free PMMA, PMMA-perylene dye, PMMA-perylene-PA, and PMMA-perylene-CLA, respectively. The activation energies $\Delta E_{a}$ obtained from dielectric relaxation data fall in the range 0.51–0.79 eV. The nearly constant loss (NCL) and quantum mechanical tunneling mechanisms were used to explain the AC conduction mechanism. The complex impedance spectra of the polymeric sheets were clarified by parallel RC element.

## Figures and Tables

**Table 1:** Kinetic and thermodynamic parameters data of PMMA-perylene dye (I), PMMA-perylene-PA (II), and PMMA-perylene-CLA (III) polymer sheets.

Compounds	Parameter					*r*
	*E* (kJ mol^−1^)	*A* (s^−1^)	Δ*S* (J mol^−1^ K^−1^)	Δ*H* (kJ mol^−1^)	Δ*G* (kJ mol^−1^)	
I	99	3.99 × 10^8^	−82	96	138	0.9954
II	46	2.67 × 10^4^	−155	44	99	0.9943
III	65	4.05 × 10^10^	−26	54	54	0.9962

**Table 2:** Activation energy of the synthesized polymeric sheets.

Polymer Sheet Sample	Activation Energy (eV)		
	From *ε*_2_ Peak Value	From *M*_2_ Peak Value	From tan*δ* Peak Value
**Free PMMA**	0.85	0.88	0.91
**PMMA-perylene dye**	0.52	0.50	0.54
**PMMA-perylene-PA**	0.79	0.76	0.76
**PMMA-perylene-I_2_**	0.53	0.51	0.56
**PMMA-perylene-CLA**	0.74	0.76	0.74

## Data Availability

Not applicable.
